# Effects of variable frequencies of kinesthesia, balance and agility exercise program in adults with knee osteoarthritis: study protocol for a randomized controlled trial

**DOI:** 10.1186/s13063-021-05386-3

**Published:** 2021-07-21

**Authors:** Aysha I. Adhama, Mukadas O. Akindele, Aminu A. Ibrahim

**Affiliations:** 1 Department of Physiotherapy, Faculty of Allied Health Sciences, College of Health Sciences, Bayero University, Kano, P.M.B 3011, Kano, Kano State Nigeria; 2 Department of Physiotherapy, Muhammad Abdullahi Wase Teaching Hospital, Hospitals Management Board, Kano, P.M.B 3160, Kano, Kano State Nigeria

**Keywords:** Knee osteoarthritis, Exercise training, Knee stability, Pain, Physical function, Proprioception, Quality of life

## Abstract

**Background:**

Knee osteoarthritis (OA) is a common painful and disabling condition that affects older individuals. Proprioceptive training programs in the form of kinesthesia, balance and agility (KBA) exercises have been reported to be beneficial for individuals with knee OA. However, the most optimal training dosage of KBA exercises is still unclear. The aim of this study is to determine the effects of different frequencies of KBA training (i.e., twice-weekly or thrice-weekly) in adults with knee OA.

**Methods:**

A single (assessor) blind, three-arm parallel, multi-center randomized controlled trial will be conducted. One hundred twenty adults with knee OA will be recruited from four tertiary hospitals in Northwestern Nigeria and randomly assigned into one of three intervention groups; twice-weekly KBA (*n* = 40), thrice-weekly KBA (*n* = 40), and conventional physiotherapy (*n* = 40) in the ratio of 1:1:1. Participants in the conventional physiotherapy group will receive two sessions of brief patient education, and sixteen sessions of ultrasound therapy, and stretching and strengthening exercises for 8 weeks. Participants in the two different KBA groups will receive KBA training according to the designed sessions for 8 weeks in addition to the conventional physiotherapy program. All groups will be assessed pre-intervention, immediately post-intervention and at 3 months, 4 months, and 6 months post-randomization. The primary outcome will be physical function (Ibadan Knee and Hip Osteoarthritis Outcome Measure) whereas the secondary outcomes will be pain intensity (Visual Analogue Scale for pain), knee stability (Knee Outcome Survey-Activities of Daily Living Scale), proprioception (electronic goniometer), and quality of life (Osteoarthritis Knee and Hip Quality of Life Questionnaire).

**Discussion:**

The findings of this study may provide evidence on the effectiveness of KBA exercise training and the ideal number of sessions needed to achieve the highest effectiveness in adults with knee OA.

**Trial registration:**

Pan African Clinical Trials Registry (PACTR201810713260138). Registered on 28 November 2017.

**Supplementary Information:**

The online version contains supplementary material available at 10.1186/s13063-021-05386-3.

## Background

Osteoarthritis (OA) is a common chronic degenerative joint disorder that affects people particularly the older population [1]. It is considered to be a disorder of dynamic pathology typified by progressive loss of articular cartilage, subchondral bone sclerosis, cyst, and osteophyte formation [2]. The impact of OA is multifactorial including pain, reduced physical function and quality of life, as well as increased healthcare and socioeconomic cost [3–5]. OA is the leading cause of disability worldwide [6]. About 250 million people are currently affected by OA, and the burden of this disorder is expected to rise globally over the coming decades perhaps due to the combined effects of the aging and obesity epidemic, along with increasing numbers of joint injuries [3].

The knee is one of the most commonly affected joints and accounts for the majority of disability from OA particularly in the elderly [7]. Epidemiological data suggest that about 14 million US populations have symptomatic knee OA, and more than half of these individuals are less than 65 years old [8]. Although data on the prevalence of knee OA in Africa is somewhat sparse [9], numerous hospital-based studies in Nigeria have indicated that knee OA is common [10–14], with prevalence rates of 19.6–20.6% in people ≥ 40 years old [10, 11] and 16.3% in people ≤ 30 years old [11]. Moreover, female gender, advanced age, obesity, knee malalignment, previous knee injury, and decreased quadriceps strength were reported to be the common risk factors associated with knee OA in Nigeria [11, 13–15] as found in the western nations [5, 16, 17].

Knee OA poses a major health problem to the society as it accounts for more walking disability than any other condition [18]. Though to date, no specific cure for OA exists, current treatment methods including pharmacological, non-pharmacologic and surgical modalities are targeted at reducing symptoms, minimizing functional disability, and limiting disease progression [19]. Non-pharmacological methods such as education/self-management, exercise, weight loss if overweight or obese, walking aids as indicated, and thermal modalities are recommended as first-line treatment [20]. These modalities are commonly prescribed for individuals with mild to moderate knee OA.

Exercise therapy is probably the most widely prescribed intervention for knee OA. Evidence suggests that exercise therapy, even though its effects are modest, is beneficial for individuals with knee OA [21, 22], hence universally endorsed by many treatment guidelines [23–27]. The most recent systematic review and meta-analysis found high-quality evidence that land-based exercise programs provide positive benefits for pain and quality of life and moderate-quality evidence of improved functional disability in individuals with knee OA [21]. While several forms of exercise interventions for this disabling condition exist, most conventionally fall into strengthening, aerobic, flexibility and skills/balance or proprioceptive exercises [28].

Proprioceptive exercises are commonly prescribed for individuals with knee OA, with the goals of improving joint proprioceptive acuity (position sense and motion sense [kinesthesia]) and dynamic stability [29] as these components are commonly altered in these individuals [30–33]. Moreover, there is evidence to suggest that proprioceptive training performed in both weight-bearing and non-weight-bearing positions can enhance proprioceptive acuity, and reduce pain as well as functional disability in individuals with knee OA [29, 34, 35].

Kinesthesia, balance and agility (KBA) is a form of proprioceptive training that has been gaining interest among researchers in the management of knee OA and knee-related injuries. This exercise training is typically designed to improve dynamic joint stability and neuromuscular control using a series of physical activities that challenge the individual’s neuromuscular system to maintain balance and coordination [36]. KBA techniques are commonly used in the rehabilitation and prevention of knee ligamentous injury [37–40] and ankle instability [41, 42]. However, in recent years, KBA has been also applied in the management of individuals with knee OA. The benefits of this intervention in knee osteoarthritic condition were first reported in a case study of a physically active, elderly woman with bilateral knee OA [43]. After receiving a 6-week, twice-weekly KBA plus therapeutic exercise program, the patient’s symptoms resolved rapidly and was able to return to recreational sports.

So far, to our knowledge, only six randomized controlled trials (RCTs) [36, 44–48] investigated the effects of KBA exercises  among individuals with knee OA. Two trials [36, 44] applied 8 weeks, three times per week of KBA plus resistance training, and found no superior benefits of this combination on Western Ontario and McMaster Universities Osteoarthritis Index (WOMAC) pain and physical function compared to resistance training alone. A similar result was also reported in another trial [45] that applied 6–8 weeks, two times per week of KBA plus resistance training. On the contrary, superiority results were reported with 8 weeks, three times per week of KBA plus resistance training compared to resistance training alone in one [46] of the earlier trials. This is also in line with another earlier trial [47] that found higher positive effects of KBA training alone on perceived pain and functional capacity when compared to non-treatment. However, when the treatment sessions of KBA training were increased to five times per week for 4 weeks [48], no superiority effects were observed over strength training on perceived pain and most symptoms. While the results of these studies are promising, the question of what is the ideal number of sessions (dosage) of structured KBA exercises that can be expected to produce the desired therapeutic outcomes however remains to be addressed. Moreover, no study has combined or compared KBA training with a conventional physiotherapy consisting of education/self-management, thermotherapy, and stretching and strengthening exercises among individuals with knee OA despite these methods are endorsed as the first-line management [20].

It is conceivable that the small to moderate effect sizes detected in exercise-based RCTs on knee OA may be due to insufficient dose used [20]. It has recently been suggested that evidence-based proprioceptive training should consider training frequency of at least three times per week, for 30 to 40 min per session to achieve the highest effectiveness [34]. The frequency and number of sets to be performed therefore should be taken into consideration when designing proprioceptive-based training programs for knee OA individuals as these exercise programs may appear challenging especially among older individuals. Thus, this study will be conducted to determine which frequency of structured KBA exercise training (i.e., twice-weekly or thrice-weekly) will produce superior therapeutic outcomes in adults with knee OA. The primary outcome will be physical function whereas secondary outcomes will be pain intensity, knee stability, proprioception, and quality of life.

## Methods

### Study design

**Fig. 1:**
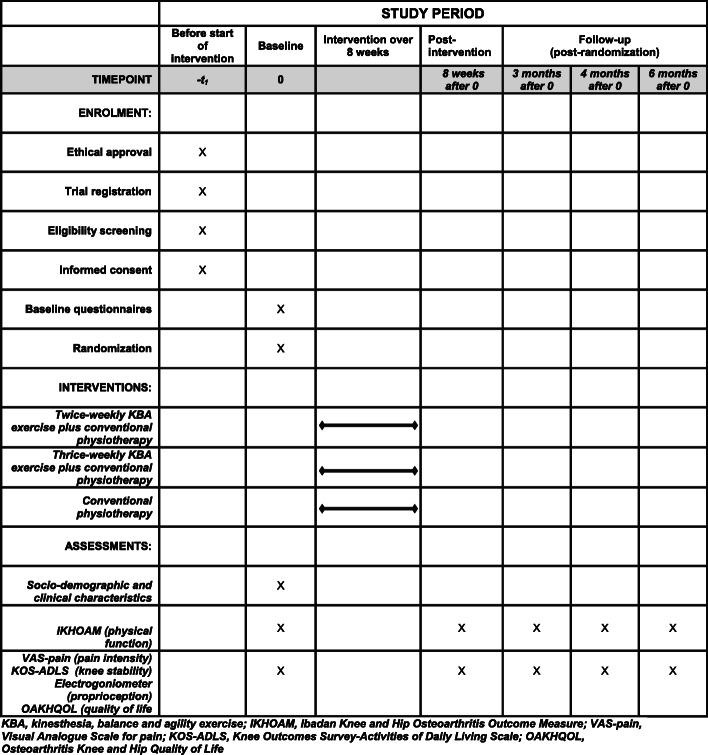
The outline of the study protocol.

This study will be a single (assessor) blind, three-arm parallel, multi-center RCT. The outline of the study protocol is shown in Fig. 1. The protocol for this study is reported in accordance with the Standard Protocol Items: Recommendations for Interventional Trials (SPIRIT) 2013 Checklist (Additional file 1).

### Study setting

The study will be conducted at four tertiary hospitals in Kano city, Kano State, Northwestern Nigeria; Muhammad Abdullahi Wase Teaching Hospital (MAWTH), Murtala Muhammad Specialist Hospital (MMSH), Aminu Kano Teaching Hospital (AKTH), and National Orthopedic Hospital Dala (NOHD). These centers were chosen to have the required number of participants.

### Training of physiotherapists/research assistants

Prior to the commencement of the study, two licensed physiotherapists with at least 2 years of clinical experience will be recruited as research assistants in each of the four hospitals. One physiotherapist will be responsible for eligibility and outcome (baseline and follow-up) assessments. This physiotherapist will be blinded to group allocation. The other physiotherapist will be responsible for treatment. All the physiotherapists will receive a 2-day training on the study procedures by the lead investigator (AIA).

### Participants’ recruitment and eligibility

Participants for this study will be consecutive patients with knee OA referred to the physiotherapy department by the general practitioners in the four tertiary hospitals. Also, recruitment adverts using local posters with the contact of the primary investigator will be pasted at various notice boards in the selected hospitals. To be eligible for the study, participants must meet the clinical criteria for the diagnosis of knee OA according to the American College of Rheumatology (ACR) criteria for unilateral or bilateral symptomatic knee OA [48] in addition to being male or female of 30 to 65 years old. They will be excluded if they have any history of knee, hip, or ankle surgery prior to the study, peripheral vascular disease, local or systemic infection, deformity in lower limbs, rheumatic disease other than OA, high-risk health status for exercise, unresolved balance or neurological disorder, and history of a lower extremity exercise program for a minimum of six weeks prior to enrollment.

### Baseline assessment

**Fig. 2:**
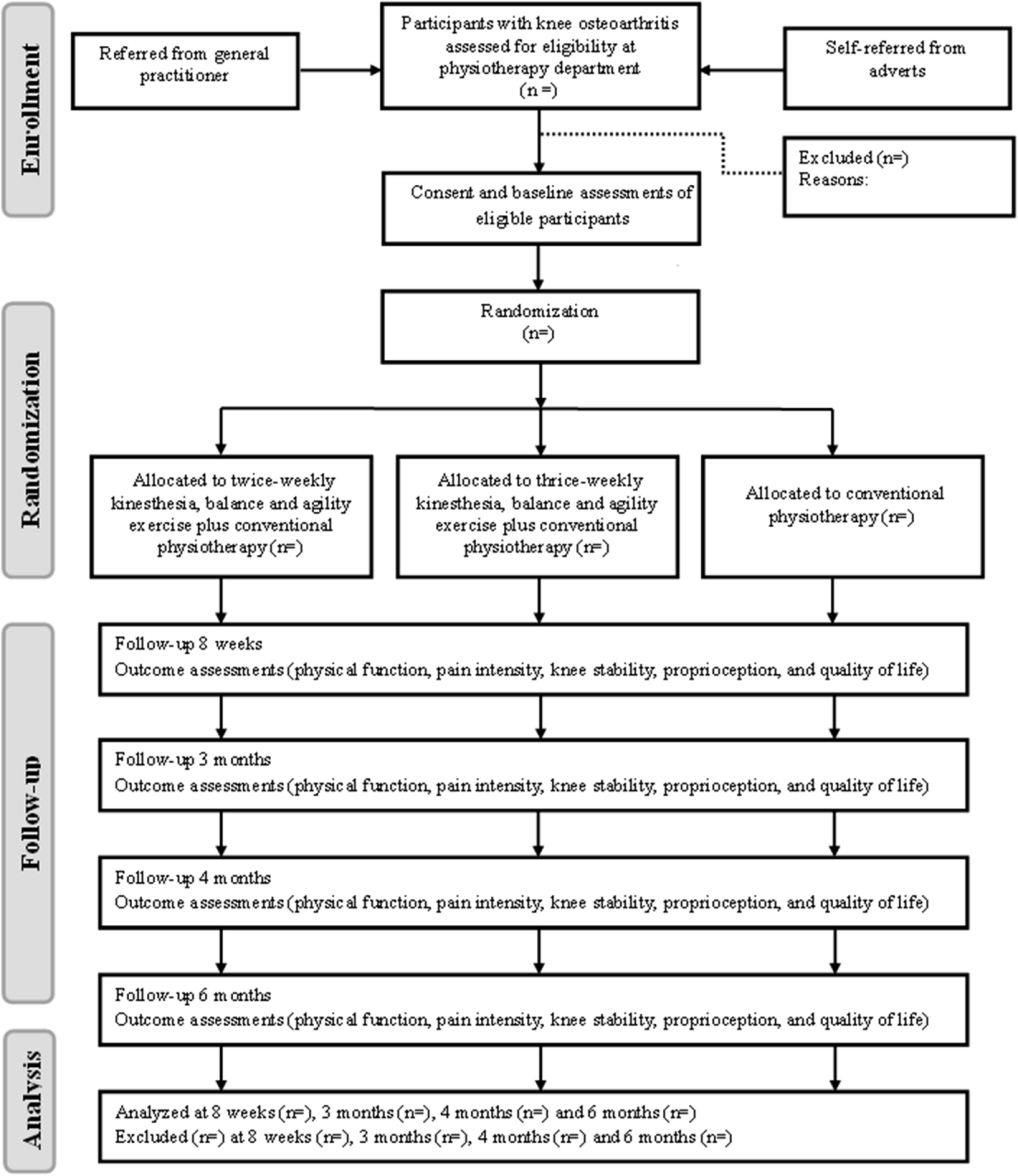
The flow of participants

 Patients will be assesed for eligibility by the physiotherapists at the four recruiting hospitals through history taking physical examination, and evaluation of self-report questionnaires. After ensuring eligibility, participants will be given oral and written information regarding the procedures and potential risks of the study. Written informed consent will be then sought and obtained. Baseline socio-demographic and clinical variables such as age, gender, marital status, education level, occupational status, height, weight, body mass index, duration of knee pain, and side affected will be collected using a prepared proforma. Baseline self-report outcome assessments will include physical function, pain intensity, knee stability, and quality of life. Baseline objective outcome assessment will be proprioception measurement. The flow of participants is depicted in Fig. 2.

### Randomization and blinding

After completing the baseline assessment, the participants will be randomized into three intervention groups; twice-weekly KBA, thrice-weekly KBA, and conventional physiotherapy (control) in the ratio of 1:1:1. The randomization will be performed using an online randomization generator (http://www.randomization.com) by utilizing block randomization with a block size of 3 and 6. A third party not  involved in the study will perform the randomization process. Allocation of participants will be concealed using consecutive numbered, sealed, and opaque envelopes. The outcome assessors (physiotherapists) will be blinded to the group allocation and will not be involved in administering the interventions. The participants will be told they are in a “twice weekly KBA” or “thrice-weekly KBA” or “conventional physiotherapy,” but the type of intervention will not be specified. Conversely, due to the nature of the interventions, it will be difficult to blind the physiotherapists providing the treatments. Unbinding conditions will be only permissible when there is a medical emergency.

### Interventions

Participants in the conventional physiotherapy group will receive brief patient education, ultrasound therapy, and stretching and strengthening exercises. The program will be delivered twice weekly for 8 weeks except for the education program which will be provided in two sessions. The stretching exercises will be performed as warm-up and cool-down. Participants in the twice-weekly KBA group will receive two sessions per week of the KBA exercise training for 8 weeks whereas participants in the thrice-weekly KBA will receive three sessions per week of the same exercise training as for the twice-weekly KBA group. Both the two KBA exercise training groups will receive the same intervention program as described for the conventional physiotherapy group prior to receiving the KBA exercise  training. All exercises will be delivered individually under the supervision of the treating physiotherapists. All lower-extremity exercises will be performed bilaterally. The participants will be instructed to perform exercises consistent with their group allocation two times per day at home to encourage self-management. They will be encouraged also to perform a walking exercise of 30 min per day at least 3 to 4 days per week at home. To enhance compliance with the home program, a leaflet with descriptions and pictures of the exercises will be provided to the participants. However, exercises that are more challenging or those requiring the strict supervision of the therapist will be not advised for the home program.

### Brief patient education

Prior to exercise  training, the participants will receive a brief education lasting approximately 15 to 20 min. The program will be delivered by the therapist responsible for exercise  training. It will be provided in a group of 3 to 5 or more participants to save time and effort. The key topics to be addressed include (1) understanding of knee OA, (2) lifestyle and physical activity/exercise, (3) diet and weight control, (4) self-management skills including active coping and pacing as well as correct use of medications (e.g., paracetamol), and (5) ergonomics and joint protection.

### Ultrasound therapy (UST)

The affected knee of the participants will be treated with UST using direct contact technique. A coupling medium (ultrasonic gel) will be used. The treatment parameters to be used include (1) frequency = 1MHz, (2) pulse width = 2.0 W/cm^2^, (3) pulse rest time = 1:1 (continuous mode), and (4) treatment time = 7 min. The UST will precede the stretching and strengthening exercises.

### Stretching and strengthening exercises

Participants will perform 3 lower-extremity stretching and strengthening exercises commonly prescribed for individuals with knee OA as described in previous studies [45, 49]. Progression for the strengthening exercises will be made by increasing the number of sets from 15 s hold to 30 s hold when the 15 s hold is no longer challenging, and the patient is performing an activity with ease and good form. However, the exercise will be discontinued if the patient reports an exacerbation of symptoms. Details of the stretching and strengthening exercises are provided in Table 1.

**Table 1:** Stretching and strengthening exercises

**Stretching exercises**			
**Exercise**	**Description**	**Intensity**	
		**Warm-up**	**Cool-down**
1.Hamstring stretch	The patient positioned supine and keeps the knees and hips at 90–90° position. The knee of one leg is then, extended progressively with the foot moving towards the ceiling until it is perpendicular to the floor. Switch sides	15 s × 6 sets	15 s × 6 sets
2.Quadriceps stretch	Patient positioned in side-lying and grasps the forefoot behind. The forefoot or ankle is pulled to a rear end. Switch sides	15 s × 6 sets	15 s × 6 sets
3.Calf stretch	The patient sits with the legs straight out in front. One leg is bent and a towel is placed around the ball of the opposite foot. While keeping the knee straight, pull the foot towards the body with the towel. Switch sides	15 s × 6 sets	15 s × 6 sets
**Strengthening exercises**			
**Exercise**	**Description**	**Intensity**	
1.Static quadriceps isometrics	The patient stands with one leg straight and the knee of the opposite leg flexed, and the foot flat on the ground. A rolled towel or a cushion is placed underneath the straight leg’s knee. The straight knee is then pressed on the towel and the position is held. Switch sides	15–30 s × 6 sets	
2**.**Seated knee extension	The patient sits on a chair with one foot flat on the floor and straightens the opposite foot. Switch sides	15–30 s × 6 sets	
3. Lying leg curl	The patient lies prone and squeezes the inner thigh to keep the legs close next to each other. The legs are then bent as far as possible by bringing the heels in towards the buttocks	15–30 s × 6 sets	

### Kinesthesia, balance and agility exercises

The exercise regimen of KBA will be done by performing a series of walking-based agility and balance exercises. The exercise protocol was adapted from previous studies [42, 44, 45] but with slight modifications regarding the training intensity and addition of visual manipulation for some selected walking-based agility exercises. The participants will perform agility exercises before proceeding to static and dynamic balance exercises. For the agility exercises, participants will begin each exercise with a walking pace of approximately 15 steps and progress to a maximum of approximately 75 steps [44, 45]. For balance training, participants will start with exercises while standing on a hard surface or floor and then proceed to exercises while standing on a form surface (Balance Soft Mat, BigMall, Nigeria). The form provides a more difficult task compared to the hard floor. Static balance exercises will be performed before progressing to dynamic balance exercises. For example, static balance will begin with a one-leg stand on a hard floor before progressing to a one-leg stand on the trainer foam [45]. The dynamic balance exercises will be progressed in the same manner but will require perturbations in different directions to be provided by the physiotherapist. The progression of each balance exercise to the next will be based on the participant’s ability by starting with the less stressful and challenging ones and increasing the number of repetitions. Participants will perform at least 3 sets of each exercise, and there will be 10 to 20 s rest between each exercise. All exercises will be progressed according to the individual’s tolerance and abilities within the structure of the program. If an exercise proved very difficult, the duration of the exercise will be adjusted based on the patient’s ability but no activity will be eliminated. However, the exercise will be discontinued if the patient reports an exacerbation of symptoms. The details of the KBA exercises are presented in Table 2.

**Table 2:** Kinesthesia, balance and agility exercises

Progression	Exercise	Treatment intensity
Weeks 1–3	Side stepping	15–75 steps × 3 sets
	Semi-tandem walk	15–75 steps × 3 sets
	Tandem walk	15–75 steps × 3 sets
	Crossbody leg swings	15–75 steps × 3 sets
	Crossover forward walk	15–75 steps × 3 sets
	Crossover backward walk	15–75 steps × 3 sets
Weeks 4–6	Toe walk	10–30 s × 3 sets
	Heel walk	10–30 s × 3 sets
	Multiple changes in direction drill (forward, backward, sideways as directed by the therapist)	10–20 s × 3 sets
	One-leg stand on a hard surface with eyes open	10–30 s × 3 sets
	One-leg stand on a hard surface with eyes closed	10–30 s× 3 sets
	Double-leg stand (eyes open) on a hard surface with perturbations	10–30 s × 3 sets
	Double-leg stand (eyes closed) on a hard surface with perturbations	10–30 s × 3 sets
Weeks 7–8	Crossover forward walk with eyes closed	15–75 steps × 3 sets
	Crossover backward walk with eyes closed	15–75 steps × 3 sets
	One-leg stand on a foam surface with eyes open	10–30 s × 3 sets
	One-leg stand on a foam surface with eyes closed	10–30 s × 3 sets
	Double-leg stand (eyes open) on a foam surface with perturbations	10–30 s × 3 sets
	Double-leg stand (eyes closed) on a foam surface with perturbations	10–30 s × 3 sets

### Outcome assessments and follow-ups

The primary outcome will be physical function whereas the secondary outcomes will be pain intensity, knee stability, proprioception, and quality of life. Similar to previous trials on knee OA, if only one knee of the patient is affected, the evaluation of the outcomes will be done for this knee. If the patient has bilateral affectation of which only one meets the ACR criterion [48], only this knee will be evaluated. However, if the patient is having bilateral affectation according to the ACR criterion, the more painful knee will be selected for outcome evaluation.

All outcomes will be assessed pre-intervention, immediately post-intervention and at 3 months, 4 months, and 6 months post-randomization. Participants missing their regular appointments will be given makeup appointments until they reach the required sessions for each group. This will be done by contacting them through phone calls. All participants will be advised to refrain from other interventions during the trial except for those taking medications as prescribed by their physician and this information will be collected.

### Physical function

The physical function of the participants will be assessed with the Hausa version of the Ibadan Knee and Hip Osteoarthritis Outcome Measure (IKHOAM) [50]. It is a 33-item instrument with 3 domains consisting of activity limitations, participation restrictions, and physical performance tests to assess physical function. Each item is rated on a Likert scale that ranges from 0 to 5. The measure is a Nigerian culture and environment-friendly clinical instrument developed for individuals with knee OA [51]. To obtain the patient’s percentage perceived level of physical function, the participant’s scores obtained are divided by the total possible score (232) and then multiplied by 100. Lower scores indicate a lower level of physical functioning [51]. The Hausa IKHOAM has been shown to have adequate internal consistency (0.64–0.95) and construct validity [50].

### Pain intensity

The Visual Analogue Scale for pain (VAS-pain) will be used to assess participants’ pain severity. It represents the intensity dimension by a 100 mm bidirectional plain line with anchor points of “no pain” (0 mm) and “worst possible pain” (100 mm) located at either end of the line [52]. Participants will be asked to rate their current level of pain by marking anywhere along the 100mm line that best indicates thier knee pain. The VAS-pain has been commonly used to evaluate pain intensity in osteoarthritic knee pain. The Hausa version of the VAS-pain has adequate alternate forms reliability (r = 0.93) [53] and construct validity [54] and will be used in this study.

### Knee stability

Participants’ subjective reports of knee stability will be measured with the Knee Outcome Survey-Activities of Daily Living Scale (KOS-ADLS). It is a 14-item questionnaire that assesses symptom-related and specific functional limitations [55]. Six items assess knee symptoms (pain, stiffness, swelling, instability, weakness, and limping), and eight items assess functional limitations (walking, stairs ascent/descent, standing, kneeling, squatting, sitting, and rising from a sitting position) experienced in the last 1 to 2 days during the performance of daily activities. Each item is scored on a 6-point Likert scale (0–5 points) [56]. The score is transformed to a 0–100 point scale with the highest score indicating the absence of symptoms and functional limitations [55]. For the purpose of this study, only the item pertaining to knee instability symptoms will be evaluated. The KOS-ADLS has been shown to have excellent internal consistency (0.92–0.93), test-retest reliability (intraclass correlation coefficient [ICC] = 0.97), and adequate construct validity [55].

### Proprioception

The participants’ knee proprioceptive range of motion will be measured using an electrogoniometer (Ergotest Technology, Norway; range: 15–320°). The use of an electrogoniometer for measuring the relative position of the knee joint has been shown to be a reliable method for assessing joint proprioception [57]. To measure the proprioceptive range of motion, the participants will be asked to wear shorts for ease of attachment of the electrogoniometer to the knee joint [58]. The participants’ eyes will be blinded to eliminate visual feedback. They will be asked to sit on a high plinth with the hip at an angle of 80° flexion in such a way that the distal hamstrings and knee joint are hanging freely at a resting position of 85° knee flexion. A thin foam will be wrapped around the tested knee to minimize cutaneous sensation feedback from the attachment of the electrogoniometer [59]. While keeping the knee in a neutral position, the electrogoniometer will be placed on the lateral side of the knee joint and its axis coinciding with the flexion/extension axis of the knee [60]. The goniometer will be kept in place using an elastic strap wrapped around the cushion padding. Participants will be instructed to bend the knee to a resting position of 85° of flexion and in this position, they will be instructed to bend from the resting position to the target angle (TA) of 70° and hold there for 5 s, then return to the resting position (85° of flexion) [61, 62]. The participants will be then asked to repeat the procedure of bending the knee from the resting position of 85° and say “YES” on perceiving they reached the target position of 70° and hold. This angle will be noted on the electrogoniometer and documented as the perceived angle (PA). The difference between TA and PA is the absolute angular error (AAE), which will be documented [61]. This procedure will be repeated three times and the average of the three readings will be calculated and recorded as the error for each participant [57]. The inter-rater (between assessors) and intra-rater (within assessors) reliability of the electrogoniometer used in the present study will be evaluated.

### Quality of life

The quality of life of the participants will be measured using the mini Osteoarthritis Knee and Hip Quality of Life Questionnaire (OAKHQOL) [63]. It is a 20-item questionnaire derived from the original 40-item questionnaire developed by Rat et al. [64]. The questionnaire consists of five dimensions subscales: physical activities, mental health, pain, social support, and social functioning; and three independent items addressing sexual life, professional life, and fear of being dependent. Each item is measured on a numerical rating scale from 0 to 10 and the mean item score becomes the corresponding dimension score [63]. The mini OAKHQOL has been shown to have adequate internal consistency (0.78–0.95), test-retest reliability (ICC = 0.66–0.89), and factorial validity in subjects with knee and hip OA [65].

As no Hausa versions of the KOS-ADLS and OAKHQOL are available for use, these questionnaires will be translated from English into Hausa language using guidelines for the process of cross-cultural adaptation of self-report measures [66]. This is to ensure that reliable data is collected and participants who are unable to complete English measures are not excluded in the trial [54]. However, English versions of IKHOAM, VAS-pain, KOS-ADLS, and OAKHQOL will be applied to those willing to respond in English.

### Adverse events

Based on the findings of our pilot study (unpublished data), no serious adverse events were reported with the KBA exercise training program. However, all participants will be informed during recruitment of the possibility of experiencing common adverse events related to exercise interventions such as muscle pull, soreness, or cramp. They will be educated that these symptoms are temporary and self-limiting. Nonetheless, in case of any serious adverse events such as exacerbating joint pain, discernible joint swelling, and excessive fatigue, they will be advised to report such events immediately to the primary investigator or treating therapists for assessment and prompt action. Any adverse events will be recorded and reported to the research ethics committee of Aminu Kano Teaching Hospital, or National Orthopaedic Hospital Dala, or Kano State Ministry of Health, Nigeria.

### Sample size estimation

Based on the results of our pilot study (unpublished data), using the effect size of 0.185 for between-group difference in the primary outcome (physical function measured by IKHOAM), a priori sample size was calculated assuming a statistical power of 90%, an alpha of 5% (two-tailed), and an effect size of 0.47. The calculations suggested a sample size of 102 would be needed. However, while anticipating a 20% attrition rate (*n* = 20), a total sample size of 120 will be needed with 40 participants per group. The calculations were performed with the G-power 3.1.9.2 software (University of Dusseldorf, Dusseldorf, Germany) [67].

### Statistical analysis

An independent statistician who will be blinded to the study procedures will conduct all statistical analyses. The normality test of the data will be verified with the Shapiro–Wilk test and visual inspection of distribution plots. To account for potential missing data in datasets, per-protocol (PP) and intention-to-treat (ITT) approaches will be considered for the data analysis. For the PP approach, only participants who completed all treatment sessions will be included in the analysis. For the ITT approach, multivariate imputation by chained equations will be employed [68]. Descriptive statistics of mean and standard deviation will be used to summarize all continuous variables while frequency and percentages will be used to summarize all categorical variables. Mixed between-within subject ANOVA will be used to assess interaction effect (group x time), main effects for time, and between-subjects effect if the data is normally distributed. However, Friedman’s ANOVA and Kruskal–Wallis test will be used to analyze within-group change and between-group difference, respectively, if the data is not normally distributed. Post hoc analysis using Bonferroni correction will be conducted for any significant between-group difference observed. Effect size will be also computed to determine the magnitude of change in outcomes. All data will be analyzed using SPSS version 23 (IBM Co., Armonk, NY, USA) at an alpha level of 0.05.

### Data management

After baseline assessment, all participants will be recognized only by their initials and numbers. Data will be stored using paper files, computer hard drive, and electronically to have a backup copy. All data values will be double-checked by assessors to check for errors and missing values before analysis.

### Dissemination

The results of this study will be submitted for publication to an international peer-reviewed journal irrespective of whether the results are positive, negative, or inconclusive.

## Discussion

There is evidence to suggest proprioceptive deficits in individuals with knee OA and that neuromuscular training programs targeting to improve proprioception are beneficial among these individuals [29–34]. The effectiveness of KBA as a proprioceptive training program in individuals with knee OA has been recently tested in clinical trials [36, 44–48], however, clinicians need to know the ideal dosage of KBA associated with the highest effectiveness as results of these previous trials were mixed. The present study will be aiming to determine the effects of a structured KBA exercise training program with different frequencies of treatment sessions (i.e., twice-weekly or thrice-weekly) on physical function, pain intensity, knee stability, proprioception, and quality of life in adults with knee OA. The outcomes will be evaluated at 8 weeks, 3 months, 4 months, and 6 months after randomization. We hope to follow-up the patients for a year if the 6-month follow-up results suggest this would be useful.

The findings of this study may provide evidence on the effectiveness of the KBA exercise training and the ideal number of sessions needed to achieve the highest effectiveness, which may guide clinical practice and minimize waste of time and resources.

Potential factors that could account for the limitations of the present study may include the lack of blinding of the treating therapists due to the nature of the interventions. Furthermore, because the present study is a multi-center trial employing the services of different treatment therapists with different skills and expertise, we cannot rule out the possibility of differential treatment. However, the assessor blind, three-arm RCT design with concealed allocation can be considered as the strengths of our study.

### Trial status

Recruitment of participants is on-going since February 2018 and is expected to be completed by June 2021 (PACTR201810713260138, registered on 28 November 2017). However, due to the COVID-19 pandemic, recruitment may not be completed until December 2021.

## Supplementary Information

**Additional file 1.** SPIRIT 2013 Checklist: Recommended items to address in a clinical trial protocol and related documents*.

## Data Availability

The full protocol for the study will be made available by the corresponding author on request.

## Abbreviations

AAE: Absolute angular error
ACR: American College of Rheumatology
AKTH: Aminu Kano Teaching Hospital
ANOVA: Analysis of variance
IKHOAM: Ibadan Knee and Hip Osteoarthritis Outcome Measure
KBA: Kinesthesia, balance and agility
KOS-ADLS: Knee Outcome Survey-Activities of Daily Living Scale
MAWTH: Muhammad Abdullahi Wase Teaching Hospital
MCID: Minimal clinically important difference
MMSH: Murtala Muhammad Specialist Hospital
NOHD: National Orthopedic Hospital Dala
PA: Perceived angle
PACTR: Pan African Clinical Trials Registry
PR: Proprioceptive
OA: Osteoarthritis
OAKHQOL: Osteoarthritis Knee and Hip Quality of Life Questionnaire
RCT: Randomized controlled trial
SPIRIT: Standard Protocol Items: Recommendations for Interventional Trials
TA: Target angle
UST: Ultrasound therapy
VAS: Visual Analogue Scale
WOMAC: Western Ontario and McMaster Universities Osteoarthritis Index
